# CNN-Optimized Electrospun TPE/PVDF Nanofiber Membranes for Enhanced Temperature and Pressure Sensing

**DOI:** 10.3390/polym16172423

**Published:** 2024-08-27

**Authors:** Ming Ma, Ce Jin, Shufang Yao, Nan Li, Huchen Zhou, Zhao Dai

**Affiliations:** 1 School of Life Sciences, Tiangong University, Tianjin 300387, China; maming@tiangong.edu.cn; 2 State Key Laboratory of Separation Membranes and Membrane Processes, Tianjin 300387, China; 231030495@tiangong.edu.cn (C.J.); 18238690219@163.com (S.Y.); zhctgu@outlook.com (H.Z.); 3 School of Chemical Engineering and Technology, Tiangong University, Tianjin 300387, China; 4 School of Chemistry, Tiangong University, Tianjin 300387, China

**Keywords:** electrospinning, PVDF nanofiber membranes, convolutional neural network, tetraphenylethylene, temperature/pressure-sensitive fluorescence

## Abstract

Temperature and pressure sensors currently encounter challenges such as slow response times, large sizes, and insufficient sensitivity. To address these issues, we developed tetraphenylethylene (TPE)-doped polyvinylidene fluoride (PVDF) nanofiber membranes using electrospinning, with process parameters optimized through a convolutional neural network (CNN). We systematically analyzed the effects of PVDF concentration, spinning voltage, tip–to–collector distance, and flow rate on fiber morphology and diameter. The CNN model achieved high predictive accuracy, resulting in uniform and smooth nanofibers under optimal conditions. Incorporating TPE enhanced the hydrophobicity and mechanical properties of the nanofibers. Additionally, the fluorescent properties of the TPE-doped nanofibers remained stable under UV exposure and exhibited significant linear responses to temperature and pressure variations. The nanofibers demonstrated a temperature sensitivity of −0.976 gray value/°C and pressure sensitivity with an increase in fluorescence intensity from 537 a.u. to 649 a.u. under 600 g pressure. These findings highlight the potential of TPE-doped PVDF nanofiber membranes for advanced temperature and pressure sensing applications.

## 1. Introduction

Temperature and pressure-sensitive materials have become indispensable in various applications such as environmental monitoring, medical diagnostics [1], and industrial process control [2]. These materials play a crucial role in providing accurate real-time environmental data, which are essential for ensuring safety, efficiency, and quality across numerous fields [3]. As the complexity and dynamism of modern technological environments increase, there is a growing demand for high-performance sensors capable of delivering precise measurements quickly and reliably [4]. This has spurred the rapid development of advanced sensors, such as thermocouple-based temperature sensors, widely utilized for their rapid response and broad operational temperature range [5]. However, their susceptibility to chemical corrosion significantly limits their applicability in extreme environments. Conversely, resistive sensors [6] offer higher mechanical reliability and long-term stability, with the flexibility to be miniaturized or shaped for various applications. Nevertheless, they generally exhibit lower sensitivity, which may not meet the demands of high-performance applications. Additionally, we noted that much of the current research in the sensor field focuses on single-factor responses (temperature, pressure, pH). Thus, developing a high-performance sensor that responds to both temperature and pressure remains a significant challenge.

Polyvinylidene fluoride (PVDF) has been widely used in sensor applications due to its excellent chemical stability, mechanical strength, and piezoelectric properties [7,8]. Its resistance to chemical corrosion and durability in harsh conditions make it a reliable material for sensors operating in extreme environments [9]. Moreover, its mechanical properties allow PVDF-based sensors to maintain performance stability under physical strain and pressure, making them suitable for pressure sensors, mechanical sensors, and accelerometers [10]. For example, research has shown that PVDF nanofibers exhibit outstanding performance in developing flexible pressure sensors and high-sensitivity chemical sensors. On the other hand, tetraphenylethylene (TPE) has attracted significant attention due to its unique aggregation-induced emission (AIE) properties [11]. AIE refers to the phenomenon where certain molecules, non-emissive in solution, become highly luminescent upon aggregation. This property was first introduced by Tang et al. [12]. As a classic AIE luminogen, TPE has been extensively studied and applied in various fields due to its remarkable emission efficiency in the aggregated state. Its response to temperature and pressure is noteworthy. At lower temperatures, TPE molecules tend to aggregate into larger polymer particles or clusters, enhancing fluorescence efficiency. In contrast, at higher temperatures, intermolecular interactions weaken, potentially leading to disaggregation or a reduction in aggregates, resulting in decreased fluorescence intensity. Additionally, elevated temperatures increase molecular thermal motion, reducing internal constraints within TPE molecules. This allows for freer rotation and vibration, further decreasing fluorescence intensity. Pressure also influences the aggregation state: high pressure can induce tighter aggregation, reducing non-radiative losses and enhancing fluorescence, while low pressure increases intermolecular distances, leading to reduced fluorescence efficiency. Incorporating TPE into PVDF can significantly enhance the sensitivity and selectivity of sensors [13], making them highly responsive to environmental changes. Studies have demonstrated that PVDF nanofibers doped with TPE exhibit enhanced sensitivity and selectivity in temperature and pressure sensing, enabling the provision of accurate real-time data even in complex environments. For instance, Ji et al. utilized glutathione (GSH)-coated copper clusters as precursors to synthesizing copper subparticles (Cu-SP), which were then used to construct and modify PVDF-HFP/CeVO_4_ NP films. Achieved by employing a catalytic hairpin assembly (CHA) strategy was the quantitative detection of miRNA-103a in the range of 100 fM to 100 nM [14]. Similarly, Ma et al. incorporated AIE-active luminogens derived from cyclopentadiene into PVDF polymers, fabricating photoluminescent electrospun nanofibers. Exhibited by these nanofibers were molecular integrity and strong, uniform phosphorescence emission with an average lifetime (τ) of 8.9 microseconds. Paved by this foundation is the way for the digital manufacturing and design of smart textiles [15].

Despite the inherent advantages of PVDF and TPE, traditional temperature and pressure sensors [16] still face significant challenges. These challenges include slow response times, large sizes, and insufficient sensitivity, limiting their effectiveness in applications requiring rapid and precise measurements. Traditional sensors often struggle to operate efficiently in complex and dynamic environments, leading to an urgent need for innovative materials that can overcome these limitations. Additionally, the manufacturing processes of these sensors typically lack the precision required to achieve consistent performance [17].

Electrospinning, as a versatile and cost-effective technique [18], offers the potential to produce nanofibers with controlled diameters [19,20] and uniform morphologies [21,22]. Studies have shown that PVDF-based nanofibers possess excellent mechanical properties and chemical stability [23], making them suitable for various sensing applications [24,25]. PVDF nanofibers have demonstrated superior performance in the development of flexible pressure sensors and high-sensitivity chemical sensors. Bhatta et al. utilized two-dimensional siloxane-polyvinylidene fluoride (S-PVDF) composite nanofiber membranes to develop a high-performance triboelectric nanogenerator (TENG) by optimizing the composition and employing electrospinning techniques. The fabricated membrane exhibited significant improvements in dielectric properties, electronegativity, and compressibility [26]. Furthermore, Xiong et al. successfully fabricated high-performance composite nanofiber membranes composed of PVDF and dopamine (DA) using electrospinning technology. These membranes feature a coherent and uniformly dispersed two-dimensional network topology, and their potential application as flexible wearable sensors for monitoring human motion and subtle physiological signals has been validated [27]. However, optimizing the electrospinning process parameters [28], such as polymer concentration, spinning voltage, tip–to–collector distance, and flow rate, remains complex.

Optimizing the electrospinning process to produce high-quality nanofiber membranes involves multiple challenges. Parameters like PVDF concentration, spinning voltage [29], tip–to–collector distance [30], and flow rate [31] significantly affect fiber morphology and diameter. The complex interactions between these parameters make single-variable optimization insufficient [32,33]. Traditional trial–and–error methods are time-consuming and inefficient, failing to capture the intricate relationships between parameters [34]. These methods typically require extensive experimentation and human resources [35], and the results lack universality [36]. Current models face limitations in accurately predicting fiber diameter and optimizing process parameters [37], failing to reflect the nonlinear effects of various parameters during electrospinning [38], leading to inconsistent fiber quality. For example, although various mathematical models have been used to predict fiber diameter [39,40,41], they often overlook the complex interactions between parameters, resulting in inadequate predictive accuracy [42]. Existing optimization methods usually rely on a large amount of experimental data, which is not only time-consuming but also costly [43].

To address these challenges, this study aims to fabricate high-performance PVDF nanofiber membranes doped with TPE using the electrospinning method. By leveraging the capabilities of convolutional neural networks (CNNs) [44], we aim to create a highly accurate predictive model to optimize electrospinning parameters [45], achieving uniform and high-quality nanofibers [46]. The study will systematically investigate the effects of PVDF concentration, spinning voltage, tip–to–collector distance, and flow rate on fiber morphology and performance [47]. Additionally, we will explore how TPE doping enhances the hydrophobicity, mechanical properties, and fluorescent characteristics of the nanofibers. The ultimate goal is to develop advanced temperature and pressure sensors with higher sensitivity, faster response times, and greater reliability, suitable for various applications. By optimizing these key parameters [48], we hope to improve the manufacturing efficiency and performance consistency of nanofibers, providing new insights and methods for the development of sensor technology [49].

## 2. Experimental Section

### 2.1. Materials and Reagents

PVDF was obtained from Jiangsu DeBao Sheng Nylon Co., Ltd. (Jiangyin, China). N, N-Dimethylformamide (DMF) was supplied by Tianjin Kermel Chemical Reagent Co., Ltd. (Tianjin, China). TPE was purchased from Shanghai McLean Biochemical Technology Co., Ltd. (Shanghai, China). All reagents were used as received without further purification.

### 2.2. Fabrication of PVDF Electrospinning Solutions and TPE-Doped PVDF Electrospinning Solutions

PVDF solutions with different mass fractions were prepared by dissolving PVDF powder in DMF. The PVDF concentrations were 15.5 wt%, 16.5 wt%, 17.5 wt%, 18.5 wt%, 19.5 wt%, and 20.5 wt%. The solutions were stirred at 80 °C for 8 h to ensure complete dissolution.

PVDF mass fraction of 17.5 wt% was selected. Using an electronic analytical balance, TPE solid powders were accurately weighed in glass bottles with stoppers, with weights of 0.0051 g, 0.0076 g, 0.0101 g, 0.0126 g, 0.0151 g, 0.0202 g, 0.0303 g, 0.0404 g, and 0.0505 g, respectively. A pipette was then used to add 5 mL of DMF to each glass bottle, preparing TPE spinning solutions with mass fractions of 0.5%, 0.75%, 1%, 1.25%, 1.5%, 2%, 3%, 4%, and 5% (referring to the mass fraction of TPE in the 17.5% PVDF spinning solution). The prepared solutions were placed in an ultrasonic cleaner for 0.5 h to ensure complete dissolution of TPE in the solvent. Using an electronic balance, 1.0023 g of PVDF solid powder was slowly added to the TPE-containing solutions. A magnetic stirrer bar was placed in each solution, and the solutions were heated at 80 °C with a stirring speed of 300 rpm for 8 h using an intelligent magnetic stirrer. After heating, the solutions were allowed to cool to room temperature and set aside for further use.

### 2.3. Fabrication of PVDF and TPE-Doped PVDF Nanofiber Membranes

The prepared PVDF electrospinning solution was transferred into a 10 mL syringe, which was connected to a 22-gauge spinning needle through a spinning connector. The syringe was fixed on a syringe pump, and the needle was connected to a high-voltage power supply. The height of the needle was set to be equal to and parallel with the collector. Under the influence of the electrostatic field, the spinning solution was ejected to form nanofibers, which were collected on the receiver after a certain period. The environmental parameters during electrospinning were primarily temperature and humidity, set at 25 ± 5 °C and 25% relative humidity, respectively. By adjusting the electrospinning parameters, nanofiber membranes under various conditions can be obtained. The nanofiber membranes were then placed in a drying oven to be dried and stored for future use.

### 2.4. Characterization

The morphology of the electrospun PVDF nanofibers was characterized using a scanning electron microscope (SEM, ZEISS Gemini SEM 500, Oberkochen, Germany). The samples were coated with a thin layer of gold before imaging. The fiber diameters were measured using Nano Measure software 1.2, and the average diameter was calculated based on the measurements of 100 fibers for each sample.

The viscosity of PVDF spinning solutions with mass fractions of 15.5 wt%, 16.5 wt%, 17.5 wt%, 18.5 wt%, 19.5 wt%, and 20.5 wt% was measured using an NDJ-79 rotational viscometer (Shanghai Changji Geological Instrument Co., Ltd., Shanghai, China). After the viscometer stabilized, data were recorded. Each measurement was repeated three times, and the average value was taken. The volume of the spinning solution was 20 mL, and the measurements were performed at room temperature (approximately 20 °C) and 15% humidity with a rotation speed of 750 r/min.

The surface tension of the spinning solutions with mass fractions of 15.5 wt%, 16.5 wt%, 17.5 wt%, 18.5 wt%, 19.5 wt%, and 20.5 wt% was measured using a DCAT21 tensiometer. The instrument was calibrated before measuring the surface tension of the spinning solutions. Each measurement was repeated three times, and the average value was taken. The measurements were conducted at a temperature of 20 °C and 15% humidity.

The conductivity of the spinning solutions with mass fractions of 15.5 wt%, 16.5 wt%, 17.5 wt%, 18.5 wt%, 19.5 wt%, and 20.5 wt% was measured using a DDS-307A conductivity meter (Shanghai Jiesheng Scientific Instrument Co., Ltd., Shanghai, China). Each solution was measured three times, and the average value was taken. The operating conditions were a temperature of 20 °C and 15% humidity.

The contact angle of the dried nanofiber membranes was measured using a JC2000DM contact angle measuring instrument. The nanofiber membranes were cut into 50 mm × 20 mm rectangles and attached flatly to glass slides with double-sided tape. The measurements were performed at room temperature (20 °C) and 15% humidity, and each sample was measured three times with the average value taken.

The mechanical properties of the PVDF membranes and PVDF membranes containing 1 wt% TPE were analyzed. The membranes were cut into 10 mm × 50 mm rectangles and placed in the jaws of a universal testing machine. The tensile speed was set to 100 mm/min. Each sample was tested three times, and the average value was used to plot the results.

The morphology and structure of the nanofiber membranes were further analyzed using SEM. The dried nanofiber membranes were cut into 10 mm × 10 mm squares, fixed on specimen stubs with conductive tape, and sputter-coated with gold for 2 min before imaging.

Fluorescence spectroscopy of the fluorescent nanofiber membranes was performed using a Shimadzu RF6000 fluorescence spectrophotometer (Kyoto, Japan). The excitation slit width was set to 5.0 nm, the emission slit width to 3.0 nm, excitation wavelength 460 nm, the scan speed to 2000 nm/min, and the sensitivity to High.

Tensile strength is a critical indicator of membrane performance. Uniaxial tensile tests were conducted using an LLY-06E electronic single-fiber testing machine with a loading speed of 5 mm/min. The tensile strength (*δ*) is defined as the tensile force (*F*) divided by the cross-sectional area (*S*) of the test sample, as shown in Equation (1).

(1) $$\delta =\frac{F}{S}$$

### 2.5. Training of Neural Network for Fiber Diameter Prediction

To predict the diameter of the PVDF nanofibers based on the electrospinning parameters, a conventional neural network (CNN) was developed and trained. The network was trained using the experimental data, which included the PVDF concentration, applied voltage, tip–to–collector distance, and flow rate as input features, and the measured fiber diameters as the output. The PVDF solution concentration ranged from 15.5 wt% to 20.5 wt%, the applied voltage ranged from 9 kV to 15 kV, the tip–to–collector distance ranged from 11 cm to 16 cm, and the flow rate ranged from 0.1 mL/h to 0.5 mL/h. The CNN architecture consisted of 16 convolutional layers and 2 pooling layers. The input image size was set to 32 × 32 pixels, with a kernel size of 3, stride of 1, and padding of 1. The input image size was set to 32 × 32 pixels, with a kernel size of 3, stride of 1, and padding of 1. The activation function used was ReLU, and the learning rate was set to 0.01. The training process involved 80 iterations. The performance of the model was evaluated using the coefficient of determination (*R*^2^) and the mean absolute error (MAE).

The PAN spinning process parameters and corresponding nanofiber diameters contained in each data group are shown in Tables S2 and S3 in the Supporting Information. The PAN spinning parameters involved, including PAN spinning solution concentration, spinning voltage, receiving distance, injection rate, and the resulting PAN nanofiber diameter, have different units, which may affect the prediction results of the CNN model and lead to significant errors. Thus, data normalization preprocessing is necessary. In this study, we employed the logarithmic function normalization method to preprocess the data, enabling all data to have the same impact scale on the model.

The network model evaluation indicators include *R*^2^ and MAE. *R*^2^ ranges from 0 to 1, and the closer *R*^2^ is to 1, the better the model’s predictive performance. MAE is used to measure the distance between the predicted values and the actual values, and the smaller the value, the better the model’s predictive performance. The mathematical expressions are as follows:

(2) $$R^{2}=1-\frac{\sum_{i=1}^{n}{\left(y_{i}-{\hat{y}}_{i}\right)}^{2}}{\sum_{i=1}^{n}{\left(y_{i}-\bar{y_{i}}\right)}^{2}}$$

(3) $$RMSE=\sqrt{\frac{1}{n}\sum_{i=1}^{n}{\left(y_{i}-\hat{y_{i}}\right)}^{2}}$$

The mathematical expression for the error σ is:

(4) $$\sigma =\frac{∆}{y_{i}}\times 100\%$$

(5) $$∆=\left|{\hat{y}}_{i}-y_{i}\right|$$

where $y_{i}$ is the actual value of the sample, ${\hat{y}}_{i}$ is the predicted value of the model, ${\bar{y}}_{i}$ is the average value of the actual values of the samples, and n represents the number of input samples.

### 2.6. Gray Value Method for Thermal Sensitivity Measurement

Photographs taken were input into a pre-written Python program, which processes them through cropping, color–to–gray conversion, and gray value reading, ultimately obtaining the gray value of each image to represent the corresponding fluorescence intensity. The experimental steps are as follows: Fluorescent nanofiber membranes were cut into 2 cm × 2 cm squares and placed on an intelligent temperature-controlled heater. Under a 365 nm UV lamp, photographs of the samples were taken with a mobile phone. The photos were first cropped, then converted to grayscale, and the gray value was read to represent the fluorescence intensity for testing thermal sensitivity. The temperature sensitivity (*S_T_*) is defined as the ratio of grayscale change to temperature change, mathematically expressed as:

(6) $$S_{T}=\frac{∆G_{V}}{∆T}$$

where Δ*G_V_* represents the change in grayscale value, Δ*T* represents the change in temperature. For membrane stability testing, the samples were photographed at room temperature, then gradually heated to 100 °C using the TM-926U intelligent temperature-controlled heater and photographed. After the temperature dropped back to room temperature, another photograph was taken. This cycle was repeated 12 times.

## 3. Results and Discussion

### 3.1. Comparison of Different Neural Network Prediction Results

**Figure 1:**
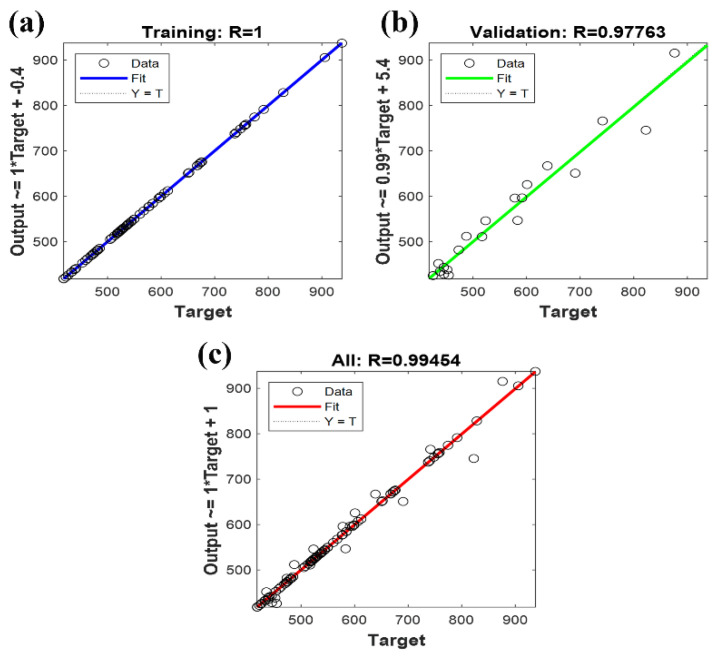
Predicted versus true values of artificial neural networks. (**a**) Training set R–value (**b**) Validation set R–value (**c**) Overall R–value.

From Figure 1, Figures S2 and S3, it is evident that among the five different artificial neural networks evaluated, the Genetic Algorithm-Extreme Learning Machine (GA-ELM) Neural Network demonstrates the closest fit between actual and predicted values, indicating superior predictive performance. The CNN showed solid predictive performance with an *R*^2^ value of 0.9776 and an RMSE of 45.4107, though its accuracy was not the highest among the networks tested. The Particle Swarm Optimization BP Neural Network (PSO-BPNN) improved upon the basic CNN, achieving an *R*^2^ of 0.9751 and a significantly lower RMSE of 17.3598, suggesting that PSO enhances the weight optimization process, reducing prediction errors. The Radial Basis Function Neural Network (RBFNN) exhibited strong performance with an *R*^2^ of 0.9757 and an RMSE of 15.9746, indicating its effectiveness in handling nonlinear relationships. The Extreme Learning Machine (ELM) network, known for its fast-training speed, achieved an *R*^2^ of 0.9886 and an RMSE of 31.3541, showing higher accuracy in capturing data variance but still having room for error minimization. The GA-ELM network outperformed all other models with an impressive *R*^2^ of 0.9959 and an RMSE of 7.446, showcasing its precision in predicting nanofiber diameters. The genetic algorithm’s optimization of the ELM significantly enhanced its predictive capability, explaining 99.59% of the variance and minimizing prediction errors. The *R*^2^ values of all models being above 0.97 indicate their effectiveness in capturing the relationship between electrospinning parameters and nanofiber diameters, while the RMSE values under 46 highlight their predictive accuracy. The GA-ELM’s superior performance, with the highest *R*^2^ and lowest RMSE, makes it the most reliable predictor among those tested. This analysis underscores the GA-ELM’s robustness and precision, making it an excellent tool for accurate nanofiber diameter predictions based on electrospinning parameters.

### 3.2. Interactions among Electrospinning Process Parameters

**Figure 2:**
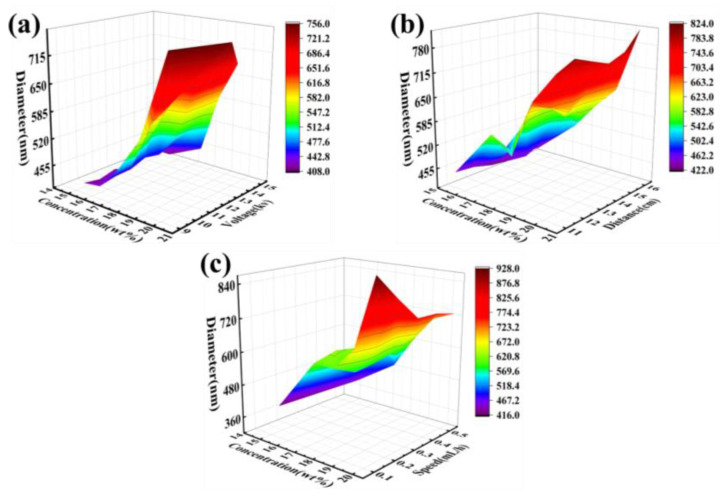
3D plots of PVDF nanofiber diameters predicted by GA-ELM model (**a**) Effect of spinning fluid concentration–voltage. (**b**) Effect of spinning fluid concentration–acceptance distance. (**c**) Effect of spinning solution concentration–injection rate.

The primary parameters influencing fiber diameter during the electrospinning process are concentration, flow rate, voltage, and tip–to–collector distance. The effects of these factors on fiber diameter were not simply linear, necessitating an exploration of how each parameter influenced the diameter. Trends in fiber diameter as influenced by concentration in conjunction with the other three parameters are discussed below. The combined effects of concentration and voltage on fiber diameter were studied with a fixed tip–to–collector distance of 13 cm and a flow rate of 0.2 mL/h. The PVDF spinning solution concentration ranged from 15.5 wt% to 20.5 wt%, with a step size of 1 wt%, and the voltage ranged from 9 kV to 15 kV, with a step size of 1 kV. As shown in Figure 2a, results indicate that as the concentration increased, the fiber diameter also increased, regardless of whether the spinning voltage was high or low. This suggests that higher solution concentrations result in thicker fibers under a constant voltage.

The interaction between concentration and tip–to–collector distance was analyzed with a fixed spinning voltage of 13 kV and a flow rate of 0.2 mL/h. The spinning solution concentration ranged from 15.5 wt% to 20.5 wt%, with a step size of 1 wt%, and the distance varied from 11 cm to 16 cm, with a step size of 1 cm. The data, presented in Figure 2b, show that the fiber diameter increased with increasing concentration when the distance remained constant. However, when the concentration was fixed, the fiber diameter first increased, then decreased, and increased again as the distance increased. This suggests a complex interaction between concentration and tip–to–collector distance, with the overall trend showing an increase in fiber diameter under these combined effects.

The effects of concentration and flow rate on fiber diameter were also examined with a fixed spinning voltage of 13 kV and a tip–to–collector distance of 13 cm. The concentration of the spinning solution ranged from 15.5 wt% to 20.5 wt%, with a step size of 1 wt%, and the flow rate ranged from 0.1 mL/h to 0.5 mL/h, with a step size of 0.1 mL/h. As shown in Figure 2c, it was observed that at lower concentrations, the fiber diameter increased with increasing flow rate. At higher concentrations, the fiber diameter first increased and then decreased with increasing flow rate. When the concentration was constant, the fiber diameter increased with increasing flow rate. This trend indicates that both concentration and flow rate significantly influence fiber diameter, with a notable interaction between the two parameters.

These analyses highlight the importance of understanding the interactions among electrospinning process parameters to optimize fiber diameter for specific applications. By exploring the combined effects of concentration with voltage, tip–to–collector distance, and flow rate, it becomes possible to fine-tune the electrospinning process to achieve desired fiber characteristics.

### 3.3. Effect of PVDF Concentration on Viscosity, Surface Tension, and Conductivity of Spinning Solutions

The primary parameters influencing fiber diameter during the electrospinning process include the viscosity, surface tension, and conductivity of the spinning solution. These properties are significantly affected by the concentration of PVDF in the solution.

**Figure 3:**
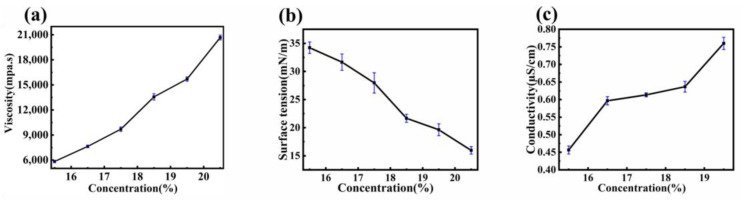
Effect of PVDF concentration on (**a**) viscosity, (**b**) surface tension, and (**c**) conductivity of spinning solutions.

Viscosity is a crucial factor in the electrospinning process, as both excessively high and low viscosities can hinder the formation of nanofibers. Generally, the viscosity of a solution is positively correlated with its concentration. As shown in Figure 3a, the viscosities of PVDF spinning solutions at concentrations of 15.5 wt%, 16.5 wt%, 17.5 wt%, 18.5 wt%, 19.5 wt%, and 20.5 wt% were 5700 mPa·s, 7500 mPa·s, 9600 mPa·s, 13,400 mPa·s, 15,600 mPa·s, and 20,500 mPa·s, respectively. The viscosity of the PVDF spinning solution increased with concentration, more than tripling as the mass fraction increased from 15.5 wt% to 20.5 wt%. This increase was mainly due to the higher probability of polymer chain entanglement in the solution as the polymer concentration increased, forming a network structure that increased flow resistance and thus the viscosity of the PVDF solution.

During the spinning process, the spinning solution pushed out from the syringe initially formed a droplet at the needle tip, known as the Taylor cone. The formation of the Taylor cone is directly related to the surface tension of the solution. When the electrostatic force is less than the surface tension of the droplet, the solution cannot be stretched to form fibers. Conversely, when the electric field strength exceeds the surface tension of the droplet, fibers can form and be collected under the electric field force. Therefore, the surface tension of the solution is critical for the formation and preparation of nanofibers. As shown in Figure 3b, as the spinning solution concentration increased from 15.5 wt% to 20.5 wt%, the surface tension decreased from 33.5 mN/m, 30.2 mN/m, 26.6 mN/m, 21.3 mN/m, 19.3 mN/m to 15.4 mN/m.

The conductivity of the spinning solution also affects the formation of multiple jets at the needle tip under a high-voltage electric field, influencing the shape of the Taylor cone. As shown in Figure 3c, the conductivity of PVDF solutions increased with increasing concentrations. When the spinning solution concentration increased from 15.5 wt% to 20.5 wt%, the conductivity increased from 0.43 µS/cm, 0.46 µS/cm, 0.48 µS/cm, 0.54 µS/cm, 0.55 µS/cm to 0.58 µS/cm.

### 3.4. Surface and Stability Properties of Nanofiber Membranes

**Figure 4:**
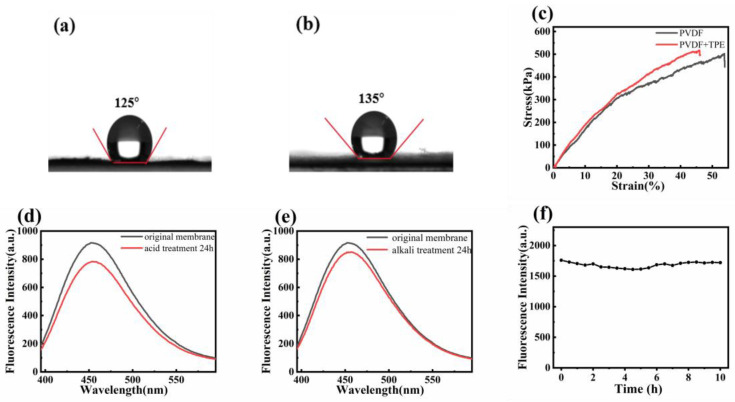
(**a**) Pure water contact angle of pure PVDF nanofiber membrane; (**b**) Pure water contact angle of TPE-doped PVDF nanofiber membrane. (**c**) Tensile stress-strain curves of pure PVDF membranes and PVDF membranes containing 1 wt% TPE. (**d**) Changes in fluorescence intensity of TPE-doped PVDF nanofiber membranes after immersion in acidic solution at pH = 1. (**e**) Changes in fluorescence intensity of TPE-doped PVDF nanofiber membranes after immersion in alkaline solution at pH = 12. (**f**) Changes in fluorescence intensity of nanofiber membranes after 10 h of continuous irradiation under a UV lamp with a wavelength of 365 nm.

PVDF is a hydrophobic polymer due to its fluorine groups (-F), while TPE is also hydrophobic as it does not dissolve in water. Figure 4a,b show the contact angles of pure PVDF nanofiber membranes and TPE-doped PVDF nanofiber membranes. The contact angle increased from 125° to 135° upon doping with TPE, indicating enhanced hydrophobicity. Figure 4c presents the tensile stress–strain curves of pure PVDF membranes and PVDF membranes containing 1 wt% TPE. The pure PVDF membrane exhibited a higher strain of approximately 53.6% but a lower stress of 500 kPa. Upon doping with 1 wt% TPE, the tensile strain decreased to about 45.8%, but the stress increased to 516.5 kPa. Doping PVDF with TPE enhances the mechanical performance of the fiber membranes by increasing their load-bearing capacity. The TPE occupies space within the nanofibers, surrounding and constraining the PVDF, which limits stretching and deformation, resulting in reduced strain but increased stress.

The chemical stability of fluorescent nanofiber membranes was evaluated by immersing them in strong acidic and alkaline solutions. Figure 4d,e show the fluorescence changes after 24 h of immersion. In Figure 4d, the fluorescence intensity of the nanofiber membrane decreased from 917 a.u. to 783 a.u. after immersion in a pH = 1 hydrochloric acid solution, representing a reduction of approximately 14%. In Figure 4e, the fluorescence intensity decreased from 917 a.u. to 852 a.u. after immersion in a pH = 12 alkaline solution, representing a reduction of approximately 7%. These results indicate that TPE-doped fluorescent nanofiber membranes exhibit good resistance to strong acids and bases. The UV stability of fluorescent nanofiber membranes was tested by continuous exposure to a UV lamp with a wavelength of 365 nm for 10 h. The fluorescence intensity at 460 nm was measured every 0.5 h. As shown in Figure 4f, the fluorescence intensity slightly decreased after 2 h of UV exposure but then increased after 2.5 h. Overall, the fluorescence intensity remained relatively stable over the extended UV exposure period, indicating that the prepared nanofiber membranes maintain their fluorescence properties even after prolonged UV exposure.

### 3.5. Morphological Analysis of Fluorescent Nanofiber Membranes

**Figure 5:**
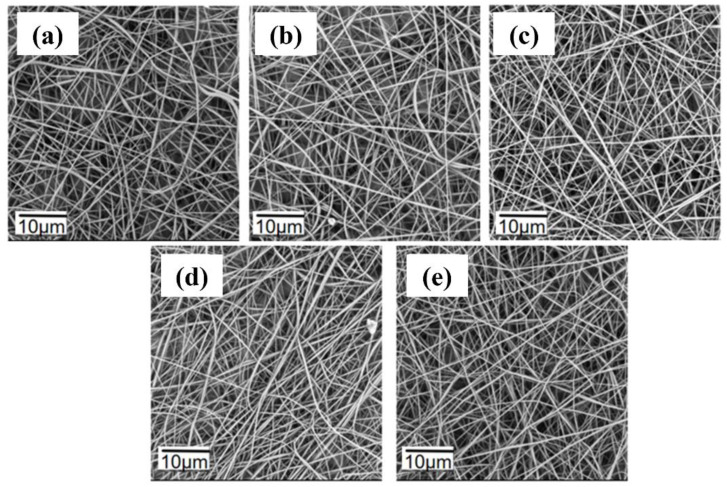
SEM images of fluorescent nanofibers at different voltages: (**a**) 9 kV; (**b**) 10 kV; (**c**) 11 kV; (**d**) 12 kV; (**e**) 13 kV.

PVDF is a hydrophobic polymer due to its fluorine groups (-F), while TPE is also hydrophobic as it does not dissolve in water. The morphology of the fluorescent nanofibers was significantly affected by the spinning voltage. The spinning parameters for 1 wt% TPE spinning solutions at different voltages are listed in Table S6 in the Supporting Information. The tip–to–collector distance was fixed at 13 cm, the flow rate at 0.2 mL/h, and the voltage varied from 9 kV to 13 kV. The PVDF concentration was 17.5 wt%, and the TPE content was 1%. As shown in Figure 5, the nanofibers exhibited uneven distribution and relatively large diameters at 9 kV and 10 kV. This was due to insufficient solvent evaporation in the jet, causing some fibers to stick together. At 11 kV, the solvent evaporated completely, resulting in smooth and uniformly distributed nanofibers, making it the optimal spinning voltage. When the voltage increased to 12 kV and 13 kV, the fiber diameters became uneven, and the morphology deteriorated. This is because higher voltages cause rapid jet ejection and quick solvent evaporation, leading to flattened fibers. Therefore, 11 kV was the most suitable spinning voltage.

**Figure 6:**
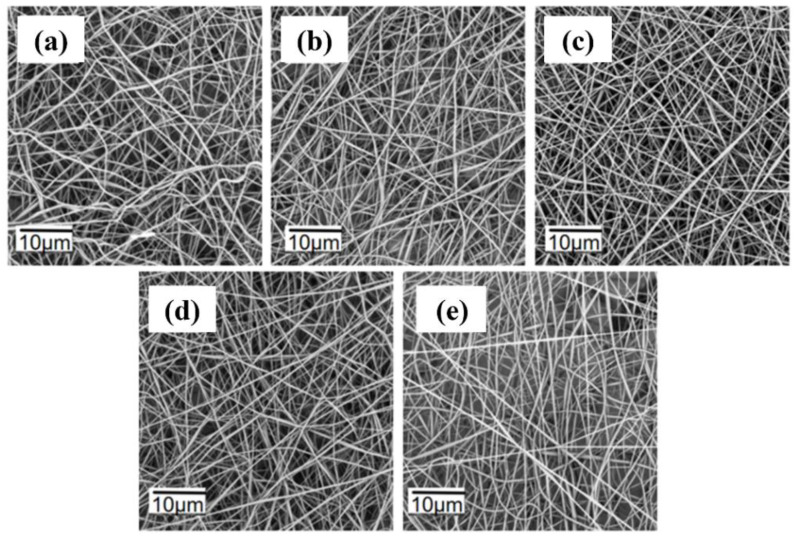
SEM images of fluorescent nanofibers at different distances: (**a**) 11 cm; (**b**) 12 cm; (**c**) 13 cm; (**d**) 14 cm; (**e**) 15 cm.

The spinning parameters for 1 wt% TPE spinning solutions at different tip–to–collector distances are listed in Table S7 in the Supporting Information. The voltage was set at 11 kV, the flow rate at 0.2 mL/h, and the distance varied from 11 cm to 15 cm. The PVDF concentration was 17.5 wt%. As shown in Figure 6, at distances of 11 cm and 12 cm, the solvent in the jet did not completely evaporate, causing fiber adhesion and a flattened morphology. The fibers were also relatively thick and poorly formed. At a distance of 13 cm, the solvent evaporated fully, resulting in well-formed, smooth, and uniformly distributed fibers. At distances of 14 cm and 15 cm, the reduced electric field strength due to increased distance led to poorer fiber uniformity. Therefore, 13 cm is the optimal tip–to–collector distance.

**Figure 7:**
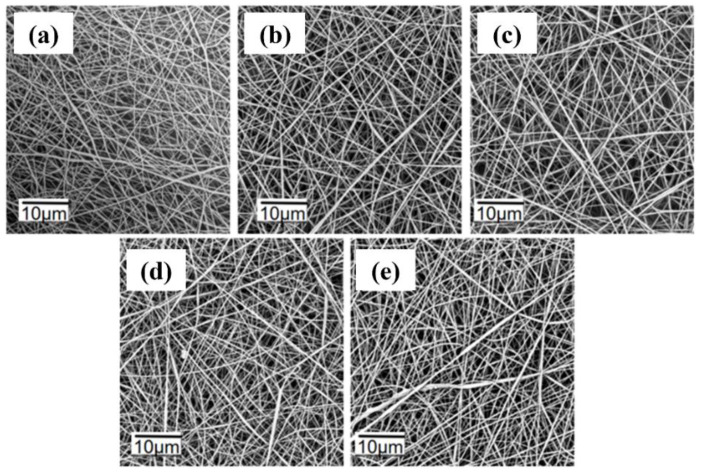
SEM images of fluorescent nanofibers at different flow rates: (**a**) 0.1 mL/h; (**b**) 0.2 mL/h; (**c**) 0.3 mL/h; (**d**) 0.4 mL/h; (**e**) 0.5 mL/h.

The spinning parameters for 1 wt% TPE spinning solutions at different flow rates are listed in Table S8 in the Supporting Information. The voltage was set at 11 kV, the tip–to–collector distance at 13 cm, and the flow rate varied from 0.1 mL/h to 0.5 mL/h. The PVDF concentration was 17.5 wt%. As shown in Figure 7, at a flow rate of 0.1 mL/h, the nanofibers were thin, unevenly distributed, and had a flattened morphology. At a flow rate of 0.2 mL/h, the jet was stable, resulting in uniformly distributed, well-formed nanofibers without adhesion. As the flow rate increased to 0.3 mL/h, 0.4 mL/h, and 0.5 mL/h, the fibers became unevenly distributed with some adhesion. This was due to the increased volume of spinning solution per unit time, which prevented timely solvent evaporation, leading to fiber adhesion. Therefore, a flow rate of 0.2 mL/h is optimal for producing uniformly distributed fibers.

### 3.6. Thermal Sensitivity Analysis of Fluorescent Nanofiber Membranes

**Figure 8:**
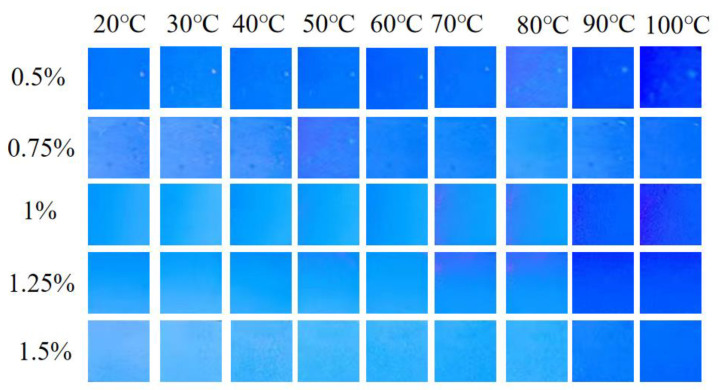
UV photographs of fluorescent nanofiber membranes with different TPE contents at different temperatures.

Fluorescent nanofiber membranes with TPE contents of 0.5%, 0.75%, 1%, 1.25%, and 1.5% were cut into suitable shapes and adhered flatly to lead blocks. The lead blocks were placed on a TM-926U intelligent temperature-controlled heater, and the temperature was gradually increased from 20 °C to 100 °C. At each temperature point, after the temperature stabilized, photographs were taken under a UV lamp. As shown in Figure 8, from top to bottom, the TPE concentrations were 0.5%, 0.75%, 1%, 1.25%, and 1.5%. From left to right, the fluorescence photographs at different temperatures from 20 °C to 100 °C are displayed. As the temperature increased, the brightness of the fluorescence decreased, with the 1 wt% TPE fluorescent nanofiber membrane showing the most significant change in brightness. Therefore, the 1 wt% TPE fluorescent nanofiber membrane was selected for thermal sensitivity studies.

**Figure 9:**
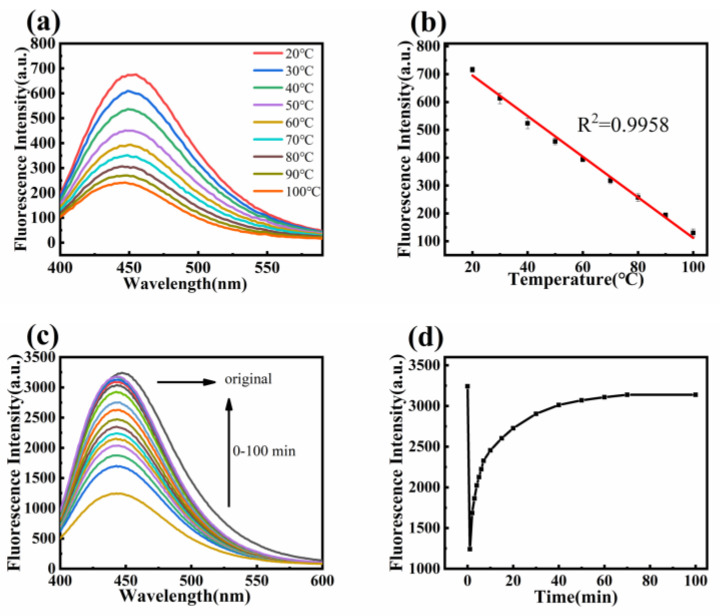
(**a**) Emission spectra of fiber membrane at different temperatures; (**b**) The fitting curve of fluorescence intensity with temperature variation; (**c**) The fluorescence intensity changes after heating the fiber membrane to 100 °C and cooling it; (**d**) The fluorescence intensity at 460 nm varies over time.

A fluorescence spectrophotometer was used to test the emission spectra of 1 wt% TPE fluorescent nanofiber membranes as the temperature increased. Figure 9a shows that as the temperature increased, the fluorescence intensity of the nanofibers decreased due to increased vibration and rotation of TPE fluorescence molecules. The fluorescence intensity at 460 nm was plotted against temperature, and Figure 9b shows that the fluorescence intensity decreased linearly with increasing temperature, with a fitting coefficient *R*^2^ = 0.9958, indicating a strong linear correlation between fluorescence intensity and temperature. The fluorescent nanofibers were first heated to 100 °C, then the heating was turned off, and the fluorescence intensity was measured at different time points. Figure 9d shows that after 80 min of cooling, the fluorescence intensity no longer changed and recovered to 95% of its original value.

Figure S8 shows that the gray value of the sample before heating was 120, and it decreased to 38 when heated to 100 °C. After cooling back to room temperature, the gray value was 100. The second heating to 100 °C reduced the gray value to 37, and cooling to room temperature resulted in a gray value of 89. After five heating–cooling cycles, the fluorescent nanofiber membrane’s properties stabilized, and the gray value no longer changed. Figure S9 also shows that initially, at room temperature, the fluorescence intensity was high, the fluorescence photo was light blue with high brightness, and the converted grayscale photo was lighter. When first heated to 100 °C, the fluorescence intensity decreased, the fluorescence photo became dark blue with low brightness, and the converted grayscale photo darkened. After cooling to room temperature, the fluorescence intensity decreased, and the colors of both the fluorescence and grayscale photos deepened compared to before heating. With more heating–cooling cycles, the color of the photos at room temperature and 100 °C gradually deepened until, after the fifth cycle, the color no longer changed.

**Figure 10:**
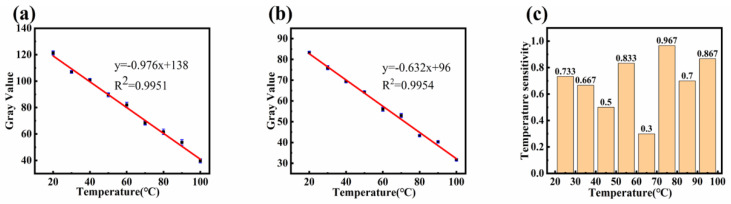
(**a**) Fitted curve of grey value versus temperature for the first heating; (**b**) Fitted curve of grey value versus temperature after five heating cycles; (**c**) Temperature sensitivity in different temperature ranges.

Using nanofiber samples that had not been heat-treated and those that had undergone five heating–cooling cycles, the temperature was gradually increased from 20 °C to 100 °C, and photographs were taken and converted to grayscale every 10 °C. Figure 10a shows the temperature fitting curve for untreated samples as the temperature changed from 20 °C to 100 °C, and Figure 10b shows the fitting curve for samples after five heating cycles. Before heating, the fitting coefficient *R*^2^ = 0.9951, and the sensitivity was −0.976 gray value/°C. After five heating cycles, the fitting coefficient *R*^2^ = 0.9954, and the sensitivity was −0.632 gray value/°C. This indicates that both untreated and heat-treated samples have a strong linear correlation between gray value and temperature, with reduced sensitivity after heating.

Table 1 compares the temperature sensitivity of the sensor developed in this work with other sensors. As shown in Figure 10c, the sensitivity was lowest in the 60–70 °C range at −0.3 gray value/°C. The highest sensitivity was in the 70–80 °C range at −0.967 gray value/°C, while the sensitivity in other temperature ranges was approximately −0.756 gray value/°C. Table 1 demonstrates that the temperature sensitivity of the sensor developed in this work was higher than that of the listed sensors, For instance, compared to AIE-PPC optical fibers, which have a temperature sensitivity of 0.01 mm/°C, our TPE-doped PVDF sensors, optimized using electrospinning parameters and convolutional neural networks, exhibit superior performance. Additionally, the sensor is smaller in size and covers a temperature range of 20–100 °C, making it more suitable for conventional temperature detection.

### 3.7. Pressure Sensitivity Analysis of Fluorescent Nanofiber Membranes

**Figure 11:**
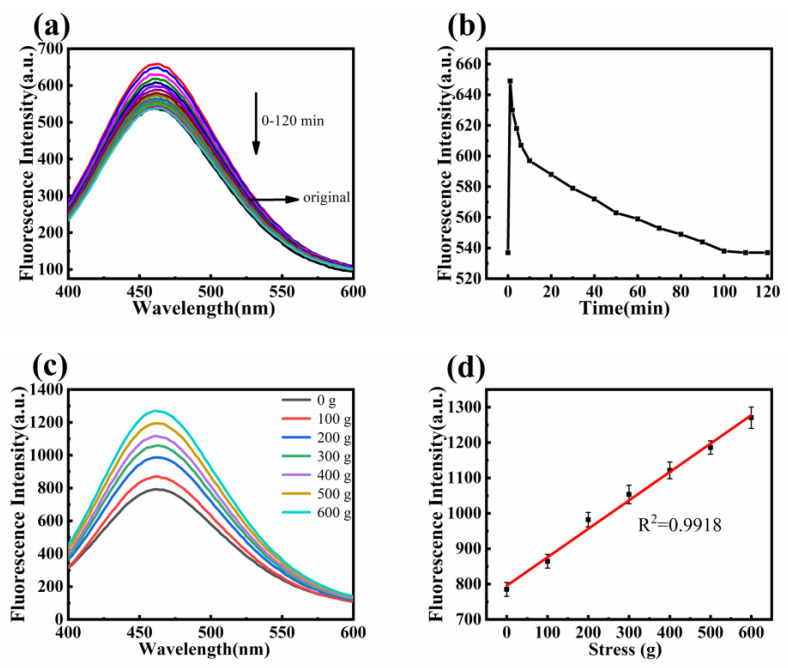
(**a**) Fluorescence recovery emission spectrum after removal of 600 g pressure; (**b**) Changes in fluorescence intensity at 460 nm over time after removal of 600 g pressure; (**c**) Emission spectra of membranes under different pressures; (**d**) Fitting curve of fluorescence intensity of fiber membrane with pressure variation.

The emission spectra of the original fluorescent nanofiber membranes were measured using a fluorescence spectrophotometer. Then, a 600 g weight was applied for 2 min, and the fluorescence intensity was measured at different time intervals. Figure 11a shows the emission spectra of the fluorescent nanofiber membrane after the removal of 600 g pressure at different times. Figure 11b shows the fluorescence intensity at 460 nm over time. Before pressure was applied, the fluorescence intensity was 537 a.u. After applying pressure, the fluorescence intensity increased to 649 a.u. due to restricted intramolecular rotation of TPE, causing most energy to be released as radiation. When the pressure was removed, the restriction on TPE molecular rotation decreased, the intramolecular kinetic energy increased, and the fluorescence intensity decreased. After 100 min, the fluorescence intensity no longer changed and recovered to 95% of the initial state.

Fluorescent nanofiber membranes with 1 wt% TPE were cut into rectangular shapes and subjected to pressures of 100 g, 200 g, 300 g, 400 g, 500 g, and 600 g for 2 min each. The emission spectra were measured using a fluorescence spectrophotometer, and the fluorescence intensity at 460 nm was plotted against pressure. Figure 11c,d show that as the pressure increases, the rotation of the TPE molecules in the nanofiber membrane is restricted, causing the energy to be released as radiation, increasing the fluorescence intensity. The fitting coefficient *R*^2^ = 0.9958, indicates a good linear correlation between fluorescence intensity and pressure.

## 4. Conclusions

In this study, we successfully prepared PVDF and TPE-doped PVDF nanofibers using the electrospinning technique and systematically investigated the effects of various parameters on fiber diameter. The GA-ELM neural network exhibited the highest prediction accuracy with an *R*^2^ value of 0.9959 and an RMSE of 7.446. Optimal conditions for uniform nanofibers were 17.5 wt% PVDF concentration, 11 kV voltage, 13 cm tip–to–collector distance, and 0.2 mL/h flow rate. TPE doping enhanced hydrophobicity, increasing the contact angle from 125° to 135°, and improved mechanical properties, increasing stress from 500 kPa to 516.5 kPa and reducing strain from 53.6% to 45.8%. The nanofibers exhibited stable fluorescence under UV light, with thermal sensitivity of −0.976 gray value/°C before heating and −0.632 gray value/°C after five cycles. Pressure sensitivity analysis showed a good linear correlation (*R*^2^ = 0.9958) between fluorescence intensity and pressure. These results highlight the significant potential of TPE-doped PVDF nanofibers in temperature and pressure sensing applications, suggesting their effective use in advanced sensors for environmental monitoring, medical diagnostics, and industrial process control.

## Figures and Tables

**Table 1:** Comparison of temperature sensitivity of various sensors.

Component Materials	Structure	Average Particle Size	Temperature Measurement Method	Temperature Range	Sensitivity
SiO_2_	Arrays	220 µm	Ratio of FL	100–800 °C	0.15 nm/°C [50]
Er^3+^-Silica	Core–shell	4 µm	Emission intensity	4–204 °C	−0.53/K [51]
Y_2_O_3_@Er^3+^/Yb^3+^-Silica	Core–shell	800 µm	Current ratio	−10–60 °C	1.3%/°C [52]
ZnO-Silica	Shell–core	100 µm	Peak position	100–300 °C	0.019 nm/°C [53]
Er^3-^-Yb^3+^@ Tellurite glass	Hollow	50 µm	Ratio of FL Intensity	30–110 °C	1.11 × 10^−2^/K [54]
PS	Solid	91.7 µm	WGM Wavelength shift	20–70 °C	0.61796 nm/°C [55]
PDMS	Solid	85 µm	Laser wavelength	25–50 °C	0.47 nm/°C [56]
E-skin	Solid	3 µm	Resistive	0–40 °C	0.0127 °C^−1^ [55]
AIE-PPC	Solid	576 nm	Laser wavelength	−40–140	0.01 mm/°C [56]
This work	Solid	680 nm	Gray value	20–100 °C	−0.632 gray value/°C
This work	Solid	680 nm	FL emission value	20–100 °C	−7.3 a.u./°C

## Data Availability

The data presented in this study are available on request from the corresponding authors.

## Supplementary Materials

The following supporting information can be downloaded at: https://www.mdpi.com/article/10.3390/polym16172423/s1, Table S1: Comparison of *R*^2^ and RMSE values for different neural networks; Table S2: Electrospinning parameters of PVDF and measured fiber diameter; Table S3: PVDF nanofibre diameter data not involved in training and model-predicted diameter data (validation set); Table S4: Network Hardware Environment; Table S5: Network Software Environment; Table S6: Spinning parameters of fluorescent nanofibers at different voltages; Table S7: Spinning parameters of fluorescent nanofibers at different distances; Table S8: Spinning parameters of fluorescent nanofibers at different flow rates; Figure S1: Comparison of actual values versus predicted values for: (a) Particle Swarm Optimization BP Neural Network (PSO-BPNN); (b) Radial Basis Function Neural Network (RBFNN); Figure S2: Comparison of actual values versus predicted values for: (a) Extreme Learning Machine (ELM) Neural Network; (b) Genetic Algorithm-Extreme Learning Machine (GA-ELM) Neural Network; Figure S3: TPE Solid Powder: (a) Under daylight; (b) Under UV light; Figure S4: Solubility of Different Mass Fractions of TPE in 17.5% Spinning Solution; Figure S5: Fluorescence intensity of TPE in different water-DMF ratios: (a) Excitation spectra of the solution; (b) Fluorescence intensity at 460 nm; Figure S6: Appearance of fluorescent nanofiber membranes: (a) Under daylight lamp; (b) Under 365 nm UV lamp; Figure S7: Excitation and emission spectra of fluorescent nanofiber membranes; Figure S8: Recovery of fluorescence after heating to 100 °C: Emission spectra; Figure S9: Fluorescence photos and converted grayscale photos during heating-cooling cycles.
