# Supplementary material for: CNN-Optimized Electrospun TPE/PVDF Nanofiber Membranes for Enhanced Temperature and Pressure Sensing
Source: Polymers (Basel). 2024 Aug 27;16(17):2423. doi: 10.3390/polym16172423 (PMC11397329; doi:10.3390/polym16172423)
Supplement: Supplementary file 1 [file polymers-16-02423-s001.zip › polymers-3140813-supplementary.pdf]

Supporting Information

CNN-Optimized Electrospun TPE/PVDF Nanofiber Membranes for Enhanced Temperature and Pressure Sensing

Ming Ma [1,2], Ce Jin [2,3], Shufang Yao [2,3], Nan Li [2,4,*], Huchen Zhou [2,3] and Zhao Dai [2,3,*]

[1] School of Life Sciences, Tiangong University, Tianjin 300387, China
[2] State Key Laboratory of Separation Membranes and Membrane Processes, Tianjin 300387, China
[3] School of Chemical Engineering and Technology, Tiangong University, Tianjin 300387, China
[4] School of Chemistry, Tiangong University, Tianjin 300387, China

Table S1 Comparison of R^2 and RMSE values for different neural networks.

Neural Network Type	R^2	RMSE
Artificial Neural Network	0.9776	45.4107
PSO-BP Neural Network	0.9751	17.3598
Radial Basis Function Network	0.9757	15.9746
ELM Neural Network	0.9886	31.3541
GA-ELM Neural Network	0.9959	7.446

Table S2 Electrospinning parameters of PVDF and measured fiber diameter

Exp. No.	Concentration (wt%)	Voltage (kV)	Receiving distance (cm)	Injection speed (mL/h)	Nanofiber diameter (nm)
1	15.5	9	14	0.4	425.22
2	15.5	10	14	0.4	418.36
3	15.5	11	14	0.4	428.53

Exp. No.	Concentration (wt%)	Voltage (kV)	Receiving distance (cm)	Injection speed (mL/h)	Nanofiber diameter (nm)
4	15.5	12	14	0.4	410.23
5	15.5	13	11	0.4	443.81
6	15.5	13	12	0.4	438.81
7	15.5	13	13	0.4	424.41
8	15.5	13	14	0.4	456.78
9	15.5	13	14	0.1	418.18
10	15.5	13	14	0.2	418.05
11	15.5	13	14	0.3	419.67
12	15.5	13	14	0.5	416.25
13	15.5	13	15	0.4	423.65
14	15.5	13	16	0.4	422.28
15	15.5	14	14	0.4	433.64
16	15.5	15	14	0.4	408.47
17	15.5	15	15	0.5	395.72
18	16.5	9	14	0.4	431.13
19	16.5	9	15	0.4	462.56
20	16.5	10	14	0.4	438.8
21	16.5	10	15	0.4	468.37
22	16.5	11	14	0.4	440.81
23	16.5	11	15	0.4	453.01
24	16.5	12	14	0.4	455.35
25	16.5	12	15	0.4	478.42
26	16.5	13	14	0.4	448.36
27	16.5	13	15	0.4	471.55
28	16.5	13	15	0.2	405.16

Exp. No.	Concentration (wt%)	Voltage (kV)	Receiving distance (cm)	Injection speed (mL/h)	Nanofiber diameter (nm)
29	16.5	14	11	0.4	456.83
30	16.5	14	11	0.5	405.77
31	16.5	14	12	0.4	481.28
32	16.5	14	13	0.4	447.11
33	16.5	14	14	0.4	444.55
34	16.5	14	14	0.1	444.81
35	16.5	14	14	0.2	497.66
36	16.5	14	14	0.3	489.63
37	16.5	14	14	0.5	444.52
38	16.5	14	15	0.4	488.43
39	16.5	14	16	0.4	458.91
40	16.5	15	14	0.4	481.41
41	16.5	15	14	0.5	474.32
42	16.5	15	15	0.4	468.94
43	16.5	15	16	0.4	466.67
44	17.5	9	14	0.4	448.37
45	17.5	10	14	0.4	477.68
46	17.5	11	14	0.4	480.47
47	17.5	12	14	0.4	523.4
48	17.5	13	11	0.1	595.43
49	17.5	13	11	0.2	598.8
50	17.5	13	11	0.3	601.05
51	17.5	13	11	0.4	579.47
52	17.5	13	11	0.5	536.47
53	17.5	13	12	0.1	560.33

Exp. No.	Concentration (wt%)	Voltage (kV)	Receiving distance (cm)	Injection speed (mL/h)	Nanofiber diameter (nm)
54	17.5	13	12	0.2	558.89
55	17.5	13	12	0.3	444.96
56	17.5	13	12	0.4	519.29
57	17.5	13	12	0.5	490.31
58	17.5	13	13	0.1	507.28
59	17.5	13	13	0.2	499.65
60	17.5	13	13	0.3	488.23
61	17.5	13	13	0.4	585.6
62	17.5	13	13	0.5	574.52
63	17.5	13	14	0.1	607.32
64	17.5	13	14	0.2	627.8
65	17.5	13	14	0.3	613.56
66	17.5	13	14	0.4	612.6
67	17.5	13	14	0.5	499.07
68	17.5	13	15	0.1	587.65
69	17.5	13	15	0.2	592.12
70	17.5	13	15	0.3	523.13
71	17.5	13	15	0.4	608.75
72	17.5	13	15	0.5	565.06
73	17.5	13	16	0.1	539.65
74	17.5	13	16	0.2	517.2
75	17.5	13	16	0.3	543.3
76	17.5	13	16	0.4	520.68
77	17.5	13	16	0.5	487.53
78	17.5	14	14	0.1	537.51

Exp. No.	Concentration (wt%)	Voltage (kV)	Receiving distance (cm)	Injection speed (mL/h)	Nanofiber diameter (nm)
79	17.5	14	14	0.2	562.33
80	17.5	14	14	0.3	556.43
81	17.5	14	14	0.4	503.87
82	17.5	15	11	0.3	503.88
83	17.5	15	14	0.4	507.67
84	17.5	15	14	0.5	521.02
85	18.5	9	15	0.4	598.26
86	18.5	10	15	0.4	600.87
87	18.5	11	15	0.4	580.56
88	18.5	12	15	0.4	571.59
89	18.5	13	11	0.4	537.01
90	18.5	13	12	0.4	648.77
91	18.5	13	13	0.4	642.8
92	18.5	13	14	0.1	606.36
93	18.5	13	14	0.2	567.6
94	18.5	13	14	0.3	576.43
95	18.5	13	14	0.4	591.46
96	18.5	13	14	0.5	612.47
97	18.5	13	15	0.4	594.61
98	18.5	13	16	0.4	590.63
99	18.5	14	15	0.4	508.12
100	18.5	14	14	0.3	546.53
101	18.5	15	14	0.2	556.58
102	18.5	15	15	0.4	625.36
103	19.5	9	14	0.4	609.72

Exp. No.	Concentration (wt%)	Voltage (kV)	Receiving distance (cm)	Injection speed (mL/h)	Nanofiber diameter (nm)
104	19.5	10	14	0.4	753.94
105	19.5	11	14	0.4	671.60
106	19.5	12	14	0.4	662.43
107	19.5	13	11	0.4	689.78
108	19.5	13	12	0.4	657.36
109	19.5	13	13	0.4	638.89
110	19.5	13	14	0.4	665.83
111	19.5	13	14	0.1	685.96
112	19.5	13	14	0.2	662.90
113	19.5	13	14	0.3	696.14
114	19.5	13	14	0.5	704.89
115	19.5	13	15	0.4	660.89
116	19.5	13	16	0.4	649.76
117	19.5	14	14	0.4	667.47
118	19.5	15	14	0.4	682.02
119	19.5	15	15	0.4	702.28
120	20.5	9	15	0.4	984.4
121	20.5	10	15	0.4	1161
122	20.5	11	15	0.4	822.23
123	20.5	12	15	0.4	785.70
124	20.5	13	11	0.4	764.71
125	20.5	13	12	0.4	789.45
126	20.5	13	13	0.4	771.87
127	20.5	13	14	0.5	736.33
128	20.5	13	14	0.4	755.19

Exp. No.	Concentration (wt%)	Voltage (kV)	Receiving distance (cm)	Injection speed (mL/h)	Nanofiber diameter (nm)
129	20.5	13	14	0.3	758.57
130	20.5	13	14	0.2	839.4
131	20.5	13	14	0.1	927.55
132	20.5	13	15	0.4	773.34
133	20.5	13	16	0.4	822.58
134	20.5	14	15	0.4	756.58
135	20.5	15	15	0.5	737.4
136	20.5	15	15	0.4	821.9
137	20.5	15	16	0.5	803.66

Table S3 PVDF nanofibre diameter data not involved in training and model-predicted diameter data (validation set)

Exp. No.	Concentration (wt%)	Voltage (kV)	Receiving distance (cm)	Injection speed (mL/h)	Nanofiber diameter (nm)	Predicted diameter (nm)	Inaccuracies (%)
1	17.5	14.5	11	0.3	580.34	579.47	0.15
2	17.5	14.5	11	0.4	601.21	593.33	1.31
3	17.5	14.5	12	0.5	493.43	489.14	0.87
4	17.5	14.5	13	0.3	505.47	496.17	0.84
5	17.5	14.5	14	0.4	516.64	508.58	1.56
6	17.5	14.5	14	0.2	483.81	460.78	4.76
7	17.5	14.5	14	0.5	513.52	500.43	2.55
8	17.5	14.5	15	0.2	541.30	533.34	0.47
9	17.5	14.5	15	0.3	510.46	493.00	3.42
10	17.5	14.5	15	0.5	472.76	466.95	1.23

Table S4 Network Hardware Environment

Name	Model Number
Processor	11th Gen Intel(R) Core(TM) i5-1135G7 @ 2.40GHz
Running Memory	4G DDR4
Graphics Adapter	NVIDIA GeForce
Hard Drive Adapter	Kingston DataTraveler 2.0 USB Device

Table S5 Network Software Environment

Name	Versions
Operating System	Window 11
Anaconda	Anaconda 3-Python3.8
pip	pip 22.0.4
Tensorflow	Tensorflow 2.4.1

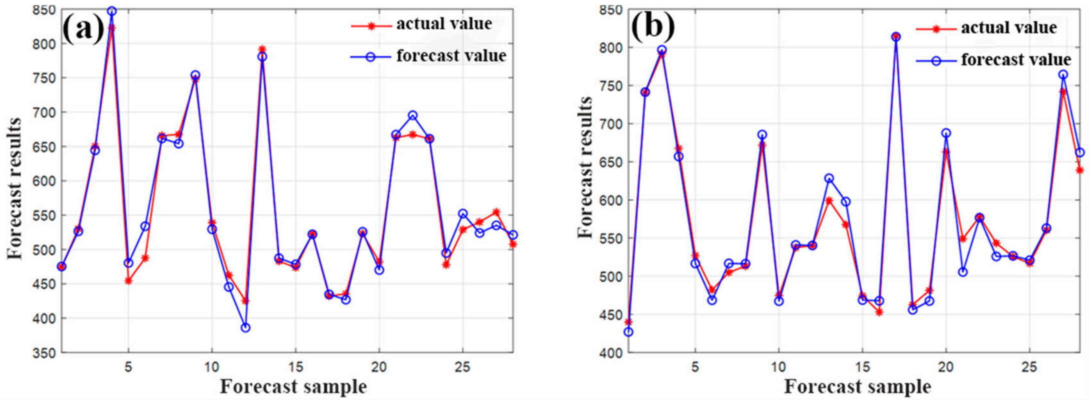

Figure S1 Comparison of actual values versus predicted values for: (a) Particle Swarm Optimization BP Neural Network (PSO-BPNN); (b) Radial Basis Function Neural Network (RBFNN).

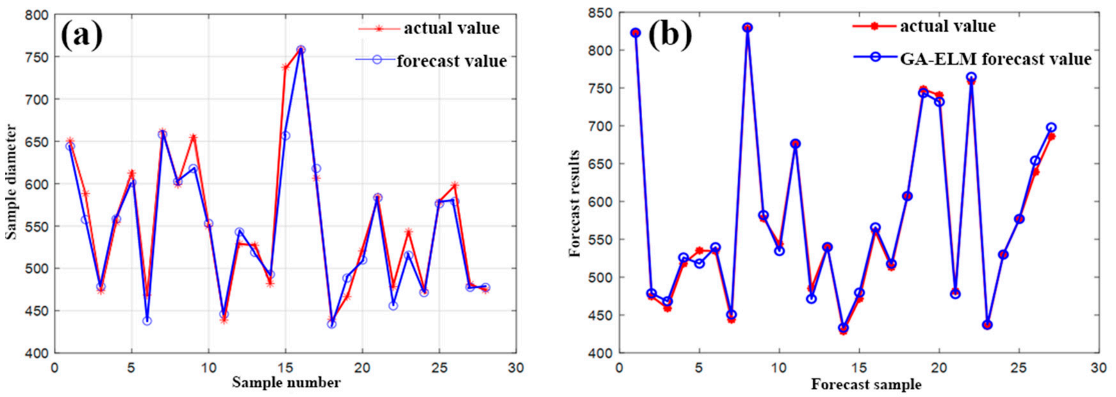

Figure S2 Comparison of actual values versus predicted values for: (a) Extreme Learning Machine (ELM) Neural Network; (b) Genetic Algorithm-Extreme Learning Machine (GA-ELM) Neural Network.

Figure S3 shows photographs of TPE powder under daylight and UV light. Under daylight, TPE appears beige, while under UV light at 365 nm, TPE emits bright blue fluorescence.

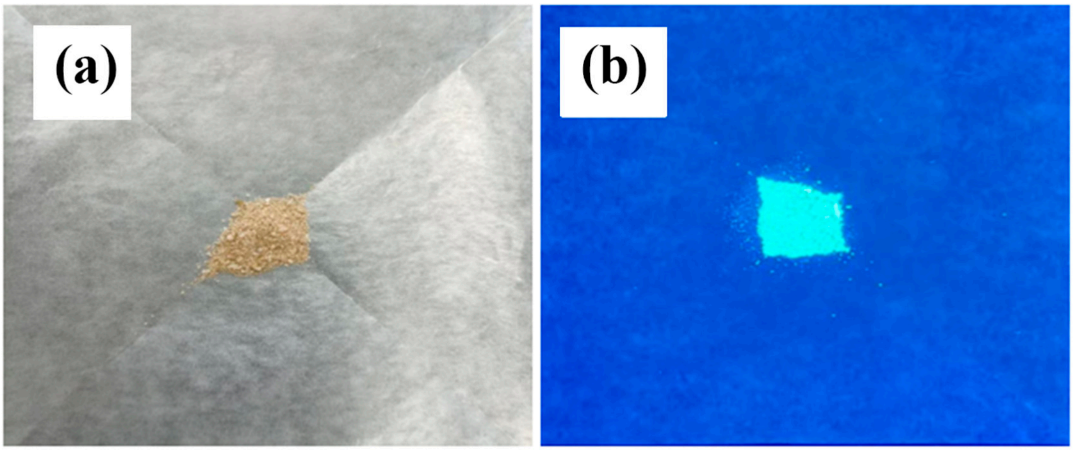

Figure S3 TPE Solid Powder: (a) Under daylight; (b) Under UV light.

Figure S4 (a) presents the solubility of TPE at mass fractions of 1%, 2%, 3%, 4%, and 5% in a 17.5% spinning solution from left to right. The (b) and (c) shows enlarged images of the bottom of the 4% and 5% spinning solutions. When the TPE mass fraction is 4% and 5%, solid precipitates at the bottom after the spinning solution is left to stand for a while. Under 365 nm UV light, the solid at the bottom emits blue fluorescence, indicating that the maximum solubility of TPE in the 17.5% spinning solution is 3%.

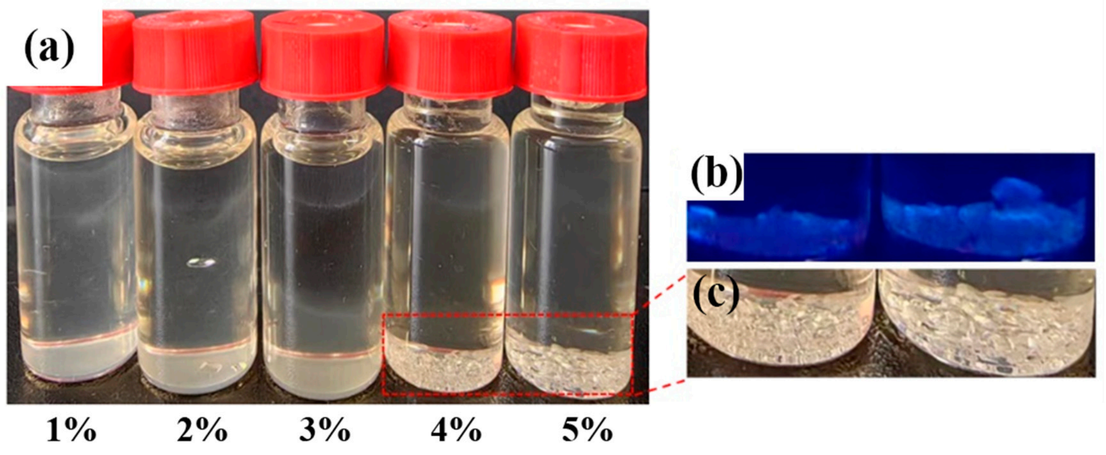

Figure S4 Solubility of Different Mass Fractions of TPE in 17.5% Spinning Solution.

Figure S5 illustrates the fluorescence intensity of TPE in solutions with varying water and DMF ratios. The fluorescence intensity initially increases with the water content, as water is a poor solvent for TPE. The TPE precipitates out, enhancing fluorescence intensity. However, excessive precipitation leads to aggregation and rapid sedimentation, reducing the TPE content in the solution and thereby decreasing the fluorescence intensity.

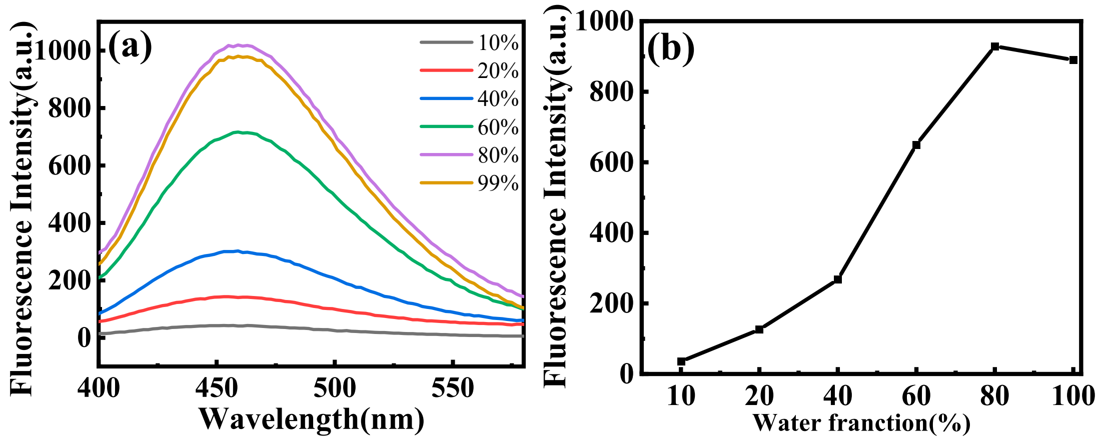

Figure S5 Fluorescence intensity of TPE in different water-DMF ratios: (a) Excitation spectra of the solution; (b) Fluorescence intensity at 460 nm.

Table S6 Spinning parameters of fluorescent nanofibers at different voltages.

TPE Content (%)	PVDF Concentration (wt.%)	Voltage (kV)	Receiving distance (cm)	Flow Rate (mL/h)
1	17.5	9	13	0.2
1	17.5	10	13	0.2
1	17.5	11	13	0.2
1	17.5	12	13	0.2

1	17.5	13	13	0.2

Table S7 Spinning parameters of fluorescent nanofibers at different distances.

TPE Content (%)	PVDF Concentration (wt.%)	Voltage (kV)	Receiving distance (cm)	Flow Rate (mL/h)
1	17.5	11	11	0.2
1	17.5	11	12	0.2
1	17.5	11	13	0.2
1	17.5	11	14	0.2
1	17.5	11	15	0.2

Table S8 Spinning parameters of fluorescent nanofibers at different flow rates.

TPE Content (%)	PVDF Concentration (wt.%)	Voltage (kV)	Receiving distance (cm)	Flow Rate (mL/h)
1	17.5	11	13	0.1
1	17.5	11	13	0.2
1	17.5	11	13	0.3
1	17.5	11	13	0.4
1	17.5	11	13	0.5

Figure S6 shows the appearance of fluorescent nanofiber membranes under a daylight lamp and a 365 nm UV lamp. In Figure S6(a), the nanofiber membrane under the daylight lamp appears uniformly distributed and defect-free, with a white color. The individual fibers are not visible due to their small diameter. In Figure S6(b), the fluorescent nanofiber membrane under the 365 nm UV lamp appears blue, which is the characteristic emission of TPE, forming a strong contrast with Figure S6(a).

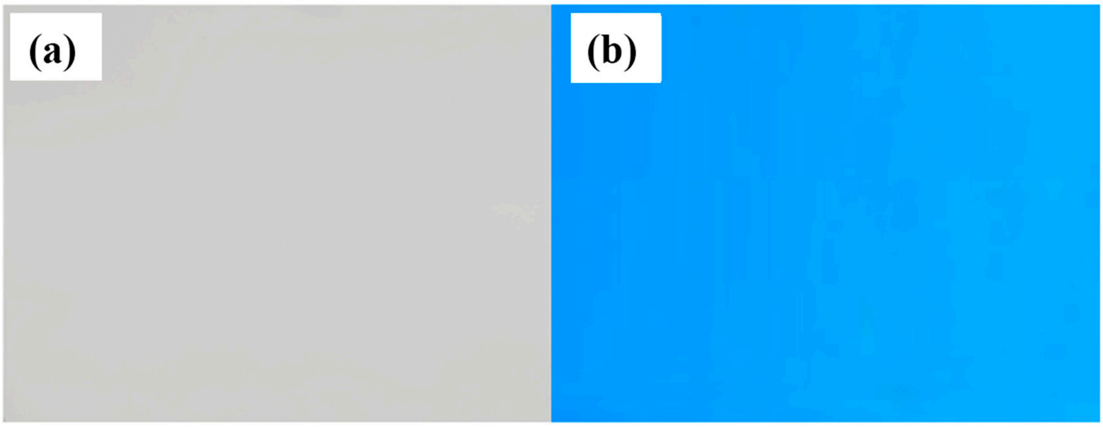

Figure S6 Appearance of fluorescent nanofiber membranes: (a) Under daylight lamp; (b) Under 365 nm UV lamp.

Figure S7 presents the excitation and emission spectra of nanofiber membranes electrospun from a spinning solution containing 1% TPE. The black curve in Figure S7 shows the excitation spectrum of the fluorescent nanofiber membrane, indicating an optimal excitation wavelength of 355 nm. The red curve represents the emission spectrum of the fluorescent nanofiber membrane, with an optimal emission wavelength of 460 nm.

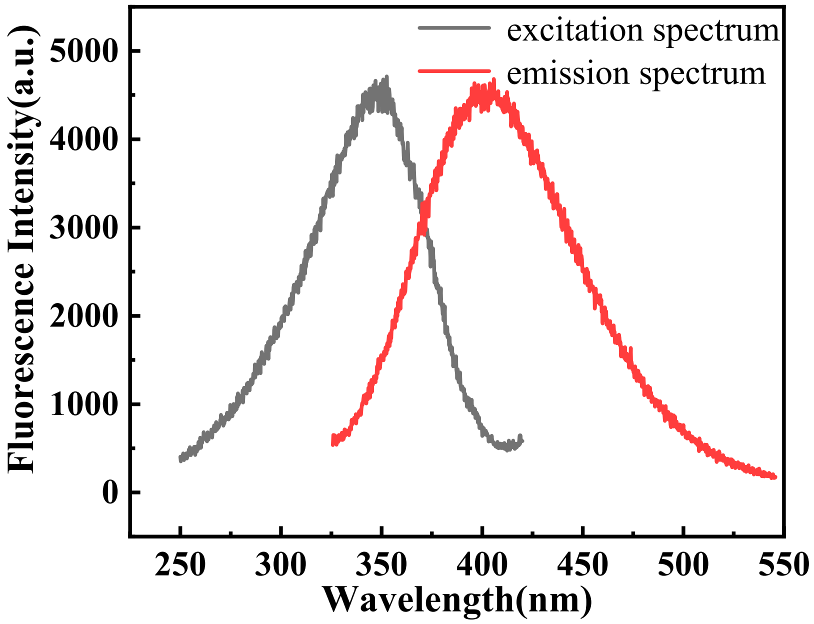

Figure S7 Excitation and emission spectra of fluorescent nanofiber membranes.

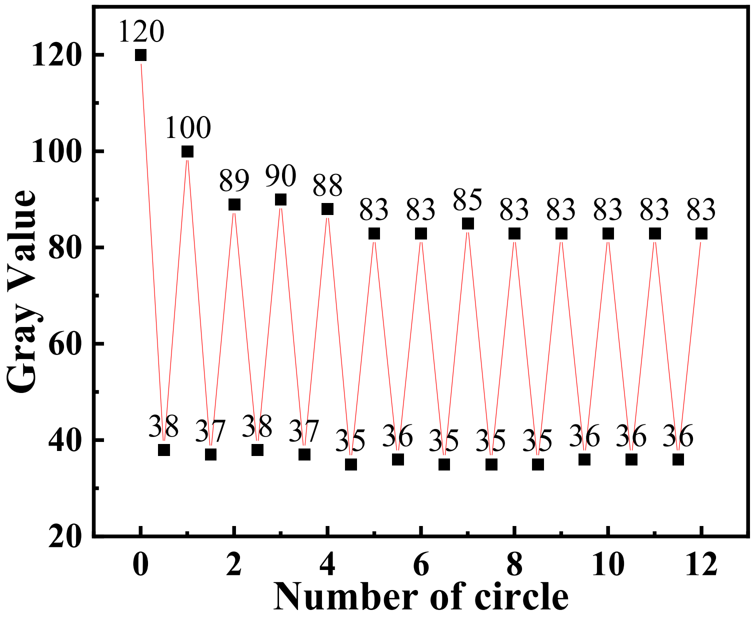

Figure S8 Recovery of fluorescence after heating to 100°C: Emission spectra.

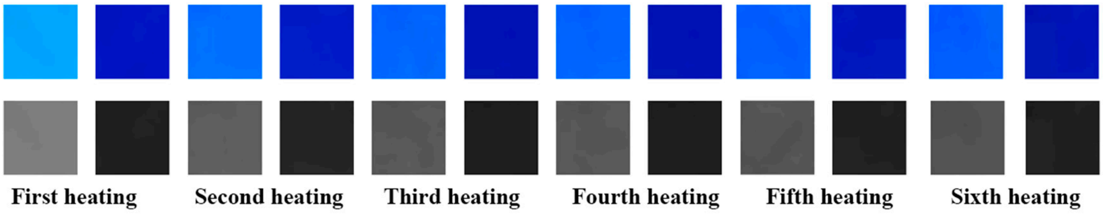

Figure S9 Fluorescence photos and converted grayscale photos during heating-cooling cycles.
